# Repetitive DNA Sequences and Evolution of ZZ/ZW Sex Chromosomes in *Characidium* (Teleostei: Characiformes)

**DOI:** 10.1371/journal.pone.0137231

**Published:** 2015-09-15

**Authors:** Priscilla Cardim Scacchetti, Ricardo Utsunomia, José Carlos Pansonato-Alves, Guilherme José da Costa Silva, Marcelo Ricardo Vicari, Roberto Ferreira Artoni, Claudio Oliveira, Fausto Foresti

**Affiliations:** 1 Universidade Estadual Paulista (UNESP), Instituto de Biociências de Botucatu/IBB, Departamento de Morfologia, Botucatu, SP, Brazil; 2 Universidade Estadual de Ponta Grossa (UEPG), Departamento de Biologia Estrutural, Molecular e Genética, Ponta Grossa, PR, Brazil; Universita degli Studi di Roma La Sapienza, ITALY

## Abstract

*Characidium* constitutes an interesting model for cytogenetic studies, since a large degree of karyotype variation has been detected in this group, like the presence/absence of sex and supernumerary chromosomes and variable distribution of repetitive sequences in different species/populations. In this study, we performed a comparative cytogenetic analysis in 13 *Characidium* species collected at different South American river basins in order to investigate the karyotype diversification in this group. Chromosome analyses involved the karyotype characterization, cytogenetic mapping of repetitive DNA sequences and cross-species chromosome painting using a W-specific probe obtained in a previous study from *Characidium gomesi*. Our results evidenced a conserved diploid chromosome number of 2n = 50, and almost all the species exhibited homeologous ZZ/ZW sex chromosomes in different stages of differentiation, except *C*. cf. *zebra*, *C*. *tenue*, *C*. *xavante* and *C*. *stigmosum*. Notably, some ZZ/ZW sex chromosomes showed 5S and/or 18S rDNA clusters, while no U2 snDNA sites could be detected in the sex chromosomes, being restricted to a single chromosome pair in almost all the analyzed species. In addition, the species *Characidium* sp. aff. *C*. *vidali* showed B chromosomes with an inter-individual variation of 1 to 4 supernumerary chromosomes per cell. Notably, these B chromosomes share sequences with the W-specific probe, providing insights about their origin. Results presented here further confirm the extensive karyotype diversity within *Characidium* in contrast with a conserved diploid chromosome number. Such chromosome differences seem to constitute a significant reproductive barrier, since several sympatric *Characidium* species had been described during the last few years and no interespecific hybrids were found.

## Introduction

*Characidium* is the largest genus within Crenuchidae and is widely distributed throughout the Neotropical region [1], comprising approximately 71 valid species [2]. However, the phylogenetic relationships within this genus are still poorly known, possibly because of the high number of undescribed species and the absence of derived characters [3–5].

Available cytogenetic data for *Characidium* revealed that all species show a conserved diploid number of 2n = 50 chromosomes. However, cytogenetic studies in distinct species and populations demonstrated a remarkable genomic differentiation in this group, like the differential distribution of repetitive sequences and independent origins of B chromosomes [3,6–15].

In addition to this remarkable karyotype diversification, one single chromosomal character seems to subdivide *Characidium* into two main groups of species: *i*) those which do not exhibit heteromorphic sex chromosomes; and *ii*) those with a heteromorphic ZZ/ZW sex chromosome system with a partial or total heterochromatinization of the W chromosome [6–9,11,13–17]. Since *C*. *zebra*, a species with morphologically indistinguishable sex chromosomes, is considered a basal species in the phylogeny of this group [4], some authors proposed that the absence of sex chromosomes might be considered a basal characteristic and its origin might have occurred once in the evolution of this genus [12,15,17]. After this origin, the differential accumulation of repetitive DNA sequences like microsatellites and ribosomal genes seem to play an important role in the diversification of the ZW sex chromosome system [6–11,13–17] while other sequences such as those that codify for the U2 spliceosomal snRNA has never been mapped, although some studies have already shown their role in the diversification of sex chromosomes in different organisms [18,19].

Although *Characidium* might be considered an excellent model for cytogenetic studies, only 17 *Characidium* species had their karyotypes studied until now. Moreover, one must note the greater concentration of analyzed species in the South/Southern region of Brazil, notably in the Upper Paraná River and East Coast River basins [14,15]. Thus, aiming to characterize the karyotypes of novel species and to investigate the patterns of repetitive DNAs distribution and their role in sex chromosomes evolution in this group, we analyzed and compared the karyotypes of 13 species of *Characidium* using classical and molecular cytogenetic tools, including conventional Giemsa staining, C-banding, chromosome mapping of ribosomal sites and U2 snRNAs genes and chromosome painting using a microdissected W-specific probe (CgW, henceforth) [12]. Results obtained here were compared with available data and provided novel information about the karyotype diversification in *Characidium*, the genome organization and evolutionary dynamics of repetitive families and their role in the sex chromosomes differentiation.

## Material and Methods

### Ethics Statement

All samples were collected in accordance with Brazilian environmental protection legislation (collection permission MMA/IBAMA/SISBIO—number 3245), and the procedures for sampling, maintenance and analysis of the specimens were performed in compliance with the Brazilian College of Animal Experimentation (COBEA) and was approved (protocol 595) by the BIOSCIENCE INSTITUTE/UNESP ETHICS COMMITTEE ON USE OF ANIMALS (CEUA).

### Sampling and Preparation of Mitotic Chromosomes

Thirteen fish species belonging to the genus *Characidium* (*C*. cf. *zebra*, *C*. *tenue*, *C*. *xavante*, *C*. *stigmosum*, *Characidium* sp.1, *Characidium* sp.2, *Characidium* sp.3, *Characidium* sp.4 and *Characidium* sp.5, *C*. *vestigipinne*, *C*. *rachovii*, *C*. *orientale* and *Characidium* sp. aff. *C*. *vidali*), captured in different South American river basins were analyzed (Table 1).

**Table 1:** *Characidium* species analyzed in the present study.

Species	N	Coordinates	Locality
*C*. *vestigipinne*	7♀ 6♂	S 28°08’03” W 52°18’40,7”	Caraguatá River (headwater)—Coxilha—RS—Brazil
*C*. *rachovii*	3♀ 7♂	S 32°05’24,1” W 52°15’09"	Arroio Cabeças Stream—Rio Grande—RS—Brazil
*C*. *orientale*	3♀ 2♂	S 32°11’26” W 52°59’30”	Chasqueiro Stream—Arroio Grande—RS—Brazil
*Characidium* sp. aff. *C*. *vidali*	8♀ 3♂	S 22°28’51,8” W 42°23’39”	Bananeiras Stream, São João River basin, Silva Jardim—RJ—Brazil
*Characidium* sp1	3♀ 2♂	S 14°36’12,8” W 57°40’51,8”	Russo River–Paraguay River basin- MT—Brazil
*Characidium* sp2	2♀ 2♂	S 14°20’33,12” W 57°31’25,7”	Vermelho River–Paraguai River basin—Tangará da Serra—MT—Brazil
*Characidium* sp3	2♀ 2♂	S 13°48’03” W 56°01’38”	Arinos River–Amazonian River basin—Nova Mutum—MT—Brazil
*Characidium* sp4	3♀ 2♂	S 3°46’37,2” W 73°20’09,37”	Nanay River—Bacia Amazonian River basin—Iquitos—Peru
*Characidium* sp5	3♀ 2♂	S 28°08’01,8” W 55°13’57”	Canoinha Stream—Between Pirapó and São Nicolau—RS—Brazil
C. cf. zebra	2♀ 3♂	S 14°38’16” W 57°09’03,52”	Duas Antas Stream—Paraguai River basin—Tangará da Serra—MT—Brazil
*C*. *tenue*	9♀ 2♂	S 33° 41’22,6” W 53°26’22,3”	Chuí Stream, Chuí River basin, Chuí—SC—Brazil
*C*. *xavante*	8♀ 2♂	S 14°25’33,8” W 54°00’56,6”	Tributary of Xingu River–Paranatinga—MT—Brazil
*C*. *stigmosum*	6♀ 9♂	S 13°45’18,5” W 47°27’20”	Tributary of Ave Maria River, Tocantins River basin, Cavalcante—GO—Brazil

Mitotic chromosome preparations were obtained from cell suspensions of the anterior portion of the kidney, according to Foresti et al. [20] and C-banding technique was performed following the protocol described by Sumner [21]. The chromosomes were classified as metacentric (m), submetacentric (sm), subtelocentric (st) and acrocentric (a) [22]. The sex of the analyzed animals was determined by visual inspection of the dissected gonads under light microscopy. The individuals were fixed in 10% formaldehyde, conserved in 70% ethanol and, after identification, deposited in the fish collection of *Laboratório de Biologia e Genética de Peixes* (LBP), UNESP- Botucatu, São Paulo, Brazil (Table 1).

### Probes and FISH experiments

Genomic DNA from *C*. *pterostictum* was extracted using the Wizard Genomic DNA Purification Kit (Promega). Partial sequences of minor and major ribosomal genes and U2 snDNA were obtained by polymerase chain reaction (PCR). The 5S and 18S rDNA sequences were amplified using the primers 5S-A (5’-TAC GCC CGA TCT CGT CCG ATC-3’) and 5S-B (5’-CAG GCT GGT ATG GCC GTA AGC-3’) [23], and NS1 (5’-GTA GTC ATA TGC TTG TCT T-3’) and NS8 (5’-TCC GCA GGT TCA CCT ACG GA-3’) [24], respectively. The U2 snDNA sites were amplified using the primers U2F (5’-ATC GCT TCT CGG CCT TAT G-3’) and U2R (5’-TCC CGG CGG TAC TGC AAT A-3’) [18]. The reactions were carried out with a final volume of 25ul, with 200uM of dATP, dCTP and dGTP, 120uM of dTTP and 80uM of digoxigenin-11-dUTP or biotin-16-dUTP; 1,5mM of MgCL_2_; 1X Buffer Taq DNA polymerase; 0,2uM of each primer; 50ng of genomic DNA; 0,5U of Taq polymerase (Invitrogen). The PCR conditions included an initial denaturation at 94°C for 5min and 32 cycles at 94°C (30s), 53–58°C (45s), and 72°C (45s), plus a final extension at 72°C for 8min. The 18S rDNA and U2 snDNA probes were labeled with digoxigenin-11-dUTP (Roche) and the 5S rDNA was labeled with biotin-16-dUTP (Roche).

The W-specific probe isolated from *C*. *gomesi* was also used and named CgW. This probe was obtained by microdissection and amplified using GenomePlex Single Cell Whole Genome Amplification Kit (WGA4, Sigma). Subsequently, this library was labeled with digoxigenin-11-dUTP (Roche) using the GenomePlex WGA Re-amplification Kit (WGA3, Sigma). Notably, this same probe was described by Pansonato-Alves et al. [12].

The hybridization conditions were the same for every analyzed sample and high stringency conditions were applied following the procedures described in Pinkel et al. [25]. Briefly, slides were incubated with RNAse (50mg/ml) for 1h at 37°C, washed in 0,005% Pepsin/10mM HCl for 5min and dehydrated in ethanol series for 2min each (70%, 80% 100%). The chromosomal DNA was then denatured in 70% formamide/2xSSC for 4min at 70°C. For each slide, 30ul of hybridization solution containing 200ng of the labeled probe, 50% formamide, 2xSSC and 10% dextran sulfate was denatured for 10min at 85°C, dropped onto the slides and hybridized overnight at 37°C in a moist chamber. Post-hybridization, the slides were washed in 0,2xSSC/15% formamide for 20min at 42°C, followed by a second wash in 0,1xSSC for 15min at 60°C and a final wash at room temperature in 4xSSC/0,5% Tween for 10min. Probe detection was carried out using anti-digoxigenin-rhodamine (Roche). Chromosomes were counterstained with DAPI (4’,6-diamidino-2-phenylindole, Vector Laboratories), and FISH images were captured under an optical photomicroscope (Olympus, BX61) and processed by using the Image Pro Plus 6.0 software (Media Cybernetics).

## Results

### Karyotypes and C-banding

**Fig 1:**
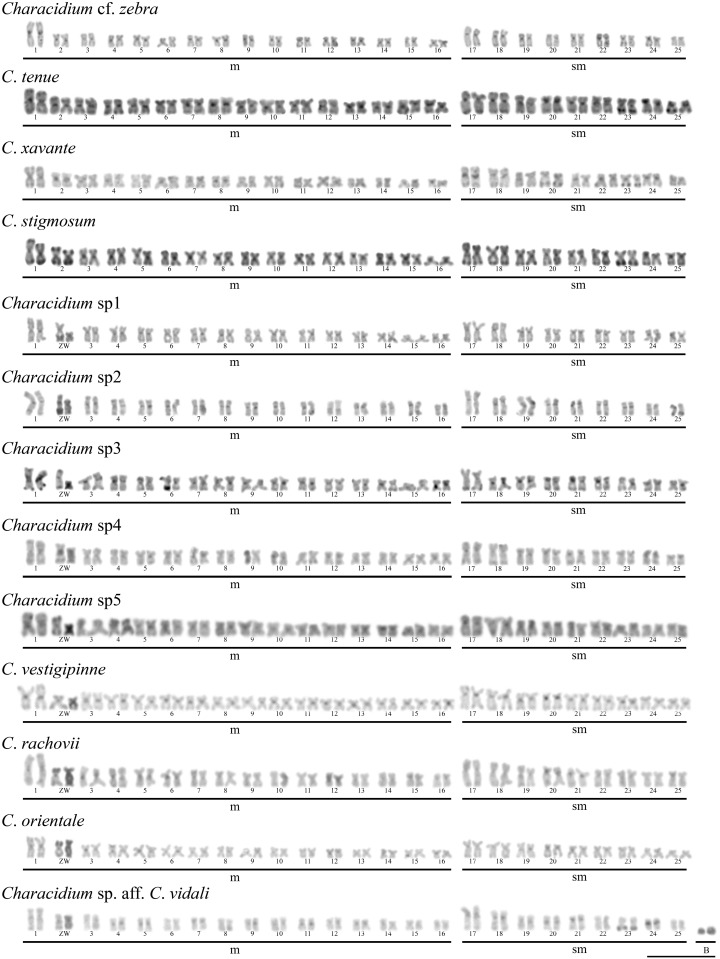
Karyotypes of *Characidium* after C-banding. Bar = 10 μm.

All species under study showed a same diploid number of 50 chromosomes, mainly composed of biarmed chromosomes (S1 Fig). In addition, specimens of *Characidium* sp. aff. *C*. *vidali* showed similar mitotically stable heterochromatic micro B chromosomes with an intrapopulational variation of 1 to 4 B chromosomes per cell. No correlation between B chromosomes and the sex of the animals was observed. C-banding revealed centromeric marks in all chromosomes in all species. In addition, conspicuous blocks of constitutive heterochromatin were detected in both Z and W chromosomes, except in *C*. cf. *zebra*, *C*. *tenue*, *C*. *xavante* and *C*. *stigmosum*, species that do not exhibit heteromorphic sex chromosomes (Fig 1).

### Mapping of repetitive DNAs

**Fig 2:**
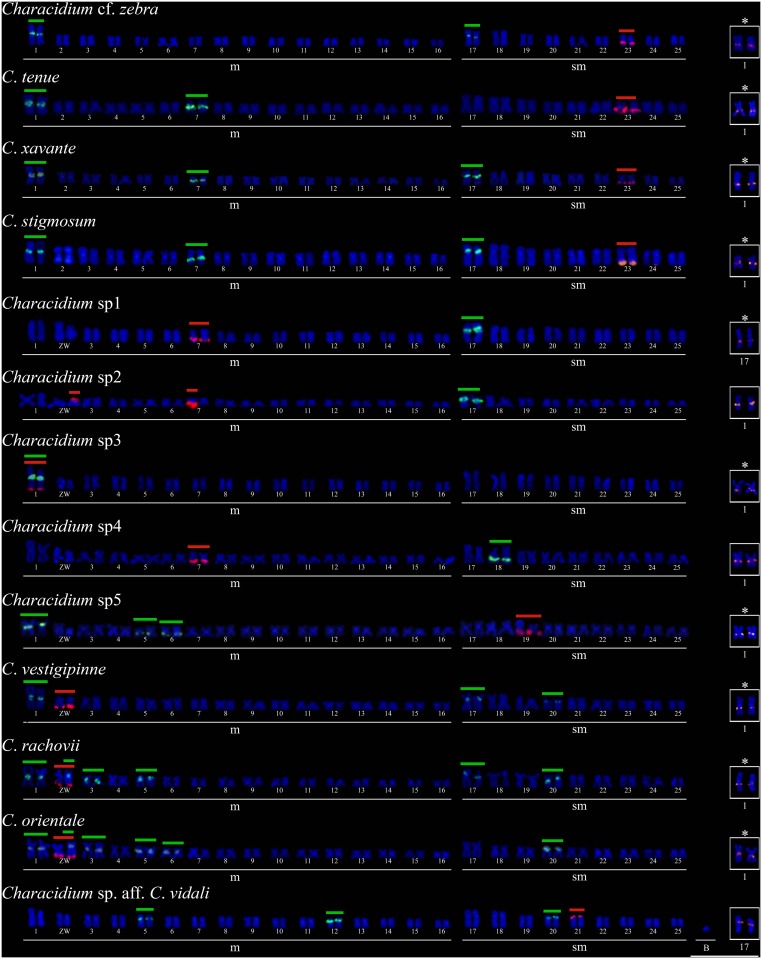
Karyotypes of *Characidium* after double-FISH with 18S rDNA probes (red), 5S rDNA (green) and snDNA U2 (in box—red). Asterisks indicate the chromosomes that harbor syntenic 5S rDNA and U2 snDNA. Bar = 10 μm.

All the analyzed species showed a single NOR-bearing pair. However, this pair was not homeologous among all the species. Thus, major ribosomal sites were located in the pair No. 23 in *C*. cf. *zebra*, *C*. *tenue*, *C*. *xavante* and *C*. *stigmosum*; in the first pair of *Characidium* sp.3; in the pair No. 7 in *Characidium* sp.1 and *Characidium* sp.4; in the pair No. 19 in *Characidium* sp.5 and pair No. 21 in *Characidium* sp. aff. *C*. *vidali*, as illustrated in Fig 2. All other species showed the 18S rDNA sites in both sex chromosomes, except *Characidium* sp.2, which presented ribosomal clusters in the W chromosome and in one of the homologues of pair No. 7 (Fig 2). Notably, we only found male specimens with one of the homologues of pair No. 7 bearing 18S rDNA sites in the latter species (data not shown).

The 5S rDNA sites distribution showed significant variation concerning the number and location of clusters. These sites were located in one chromosome pair in *Characidium* sp.1, *Characidium* sp.2, *Characidium* sp.3 and *Characidium* sp.4; in two chromosome pairs in *C*. cf *zebra* and *C*. *tenue*; and in 3 chromosome pairs in *C*. *xavante*, *C*. *stigmosum*, *C*. *vestigipinne*, *Characidium* sp.5 and *Characidium* sp. aff. *C*. *vidali*, as showed in Fig 2. In *C*. *rachovii* and *C*. *orientale*, signals of hybridization were detected in the W chromosome and in five autosomal pairs (Fig 2), while 5S rDNA sites were not detected in the Z chromosome of these species.

Finally, the cytogenetic mapping of the U2 snDNA clusters evidenced its location in the chromosomes of the first metacentric pair in almost all species, except *Characidium* sp. aff. *C*. *vidali* and *Characidium* sp.1 in which these repetitive sequences were detected in the first subtelocentric chromosome pair (Fig 2 –box).

### Chromosome painting with CgW probe

**Fig 3:**
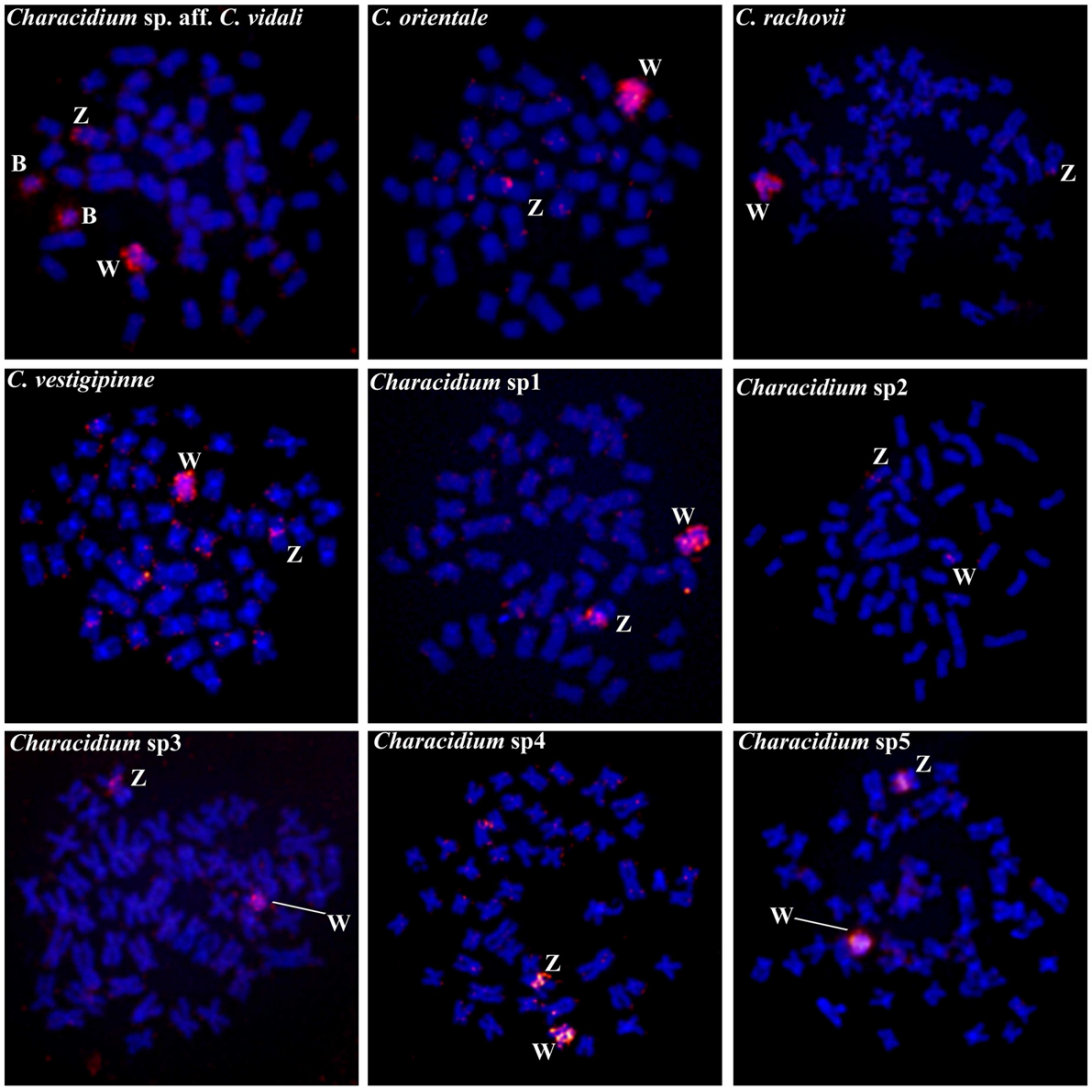
Metaphase chromosome plates after FISH with CgW probe in different *Characidium* species. The sex chromosomes are indicated. Bar = 10 μm.

The CgW probe completely painted the W chromosome of *C*. *vestigipinne*, *C*. *orientale*, *Characidium* sp1, *Characidium* sp4 and *Characidium* sp5. Additionally, this probe partial- and differentially painted the W chromosome of some species: *i*) the long arms and the centromeric region in *C*. *rachovii*; *ii*) the short arms and the centromeric region in *Characidium* sp. aff. *C*. *vidali* and *Characidium* sp3; and *iii*) the centromeric region in *Characidium* sp2 (Fig 3). Notably, the CgW probe also completely painted the B chromosomes of *Characidium* sp. aff. *C*. *vidali* (Fig 3). Conversely, this probe generated dispersed signals in several chromosomes in the species without heteromorphic sex chromosomes (S2 Fig).

**Fig 4:**
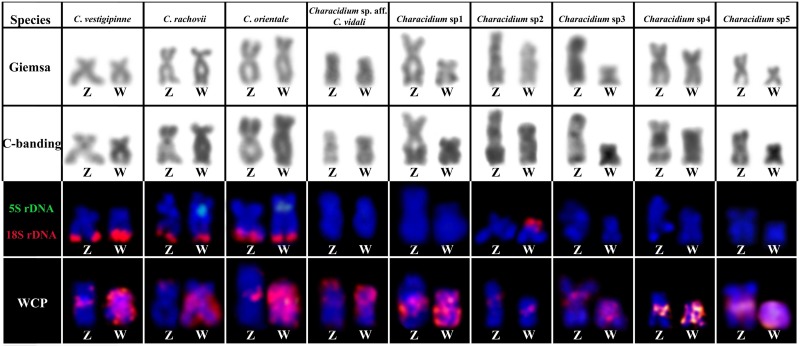
Giemsa, C-banding, FISH with the 18S rDNA probe (red), 5S rDNA (green) and with CgW probe (red) of different Z and W chromosomes of *Characidium* species. Bar = 5 μm.

Regarding the Z chromosome, the CgW probe hybridized in the proximal region of this chromosome in *C*. *vestigipinne*, *C*. *orientale*, *Characidium* sp1, *Characidium* sp2, *Characidium* sp3, *Characidium* sp4 and *Characidium* sp5. In *Characidium* sp. aff. *C*. *vidali*, faint painting signals were observed in the proximal region and strong painting signals in the subtelomeric regions of the short arm. Finally, in *C*. *rachovii*, painting signals were only observed in the subtelomeric regions of the short arm (Fig 3). All the applied techniques in the characterization of Z and W chromosomes of *Characidium* species are summarized in Fig 4.

## Discussion

### Karyotype differentiation in *Characidium*

Cytogenetic analysis was performed in 13 species of *Characidium*, 12 of these karyotypes being reported for the first time, including *Characidium vestigipinne*, *C*. *rachovii*, *C*. *orientale*, *Characidium* sp. aff. *C*. *vidali*, *C*. *tenue*, *C*. *xavante*, *C*. *stigmosum* and 5 still unidentified species, increasing the number of karyotyped species in this genus to 29 (S1 Table). Our results corroborated the general karyotypic trends for *Characidium* and evidenced very similar karyotypes for all species, mainly related to the conserved diploid chromosome number (2n = 50), the predominance of biarmed chromosomes and the similar karyotypic formulae. However, several examples of chromosome diversification in the within- and between-species level might be listed, including the interstitial telomeric sites (ITS) spreading, differential distribution of ribosomal genes and microsatellite sequences, homomorphic karyotypes or the presence of sex chromosomes in distinct stages of differentiation and the occurrence of supernumerary chromosomes in some species or populations [3,8,9,12,15,17,26].

Previous studies showed the occurrence of supernumerary chromosomes in specific populations of certain species of *Characidium*, like *C*. *pterostictum*, *C*. *oiticicai*, *C*. *gomesi* and *C*. cf. *zebra* [3,12,27]. Notably, Pansonato-Alves et al. [12] suggested that, although some of these B chromosomes share DNA sequences, like those one observed in *C*. *pterostictum* and *C*. *gomesi*, it could be supposed that they were independently originated in their respective lineages. Thus, the occurrence of multiple origins from similar chromosomes, in this case the sex chromosomes, could explain the sharing of sequences among independently originated B chromosomes.

In the present work we describe a novel case of heterochromatic B chromosomes in the species *Characidium* sp. aff. *C*. *vidali*. Interestingly, the supernumerary chromosomes of this species share sequences with the CgW probe, as in *C*. *pterostictum* and *C*. *gomesi*, pointing to sex chromosomes as the possible ancestor of these B chromosomes. In fact, similarities between B and sex chromosomes have been found in several species [28]. Since *Characidium* sp. aff. *C*. *vidali* does not seem to be morphologically related to *C*. *pterostictum* and *C*. *gomesi* [29], it can be suggested that this B chromosome system also arose independently. Considering that sex chromosomes are subjected to high rates of rearrangements and structural modifications in *Characidium* [10,14,15,17,26] it can be proposed that supernumerary chromosomes might be independently originated as by-products of such chromosomal rearrangements and then were fixed in the different species or populations. However, one must note that repetitive DNA sequences are highly dynamic and may easily spread across the chromosomes [30], particularly if these chromosomes have non-recombining genomic regions, like B and sex chromosomes. In this sense, our painting results could be reflecting the amplification of repetitive DNA sequences in the B chromosomes that are also present in other chromosomes. Thus, conclusions should be taken with caution since chromosome painting data may support a false idea of common descent between sex and B chromosomes, as stated by Pansonato-Alves et al. [12].

The cytogenetic mapping of the different types of repetitive DNA revealed distinct evolutionary dynamics. For example, it was shown that both ribosomal sites presented a variable chromosomal location among the different species. However, while the major ribosomal sites were conserved in number of clusters (2 loci per genome), the 5S rDNA was quite diverse, ranging from 2 to 10 sites. In fact, previous studies had already shown variations regarding the distribution of ribosomal sites in the within- and between-species level in this group [9,11,14,31]. Such variations were probably accumulated independently during the process of allopatric differentiation, probably due to the association with transposable elements [14]. In contrast to rDNAs, U2 snRNA genes distribution was highly conserved in this genus, always in a single chromosome pair, the first metacentric pair apparently homeologous in almost all species except in *Characidium* sp. aff. *C*. *vidali* and *Characidium* sp.1. These findings are consistent with previous studies carried out in fish and grasshoppers [18,19,32].

In addition, co-localization of U2 snDNA and 5S rDNA is also reported in two different groups of species of *Characidium*: *i*) southern species showing heteromorphic sex chromosomes (e.g. *C*. *vestigipinne* and *C*. *orientale*); and *ii*) species that do not display sex chromosomes (*C*. *zebra*, *C*. *tenue*, *C*. *xavante* and *C*. *stigmosum*). Hence, this study introduces an additional example of co-localization between U genes and ribosomal sites, a feature previously described in other organisms [33–35].

### Diversification and evolution of sex chromosomes

In this study, all the analyzed species, except *C*. cf. *zebra*, *C*. *tenue*, *C*. *xavante* and *C*. *stigmosum*, showed heteromorphic sex chromosomes in distinct stages of differentiation. Therefore, to our knowledge, a total of five species within *Characidium* do not exhibit heteromorphic sex chromosomes.

A consensus model for the origin of the heteromorphic sex chromosome system in *Characidium* proposes a single origin from a NOR-bearing autosome [10,12]. However, an intriguing feature is that *C*. *xavante* and *C*. *lagosantense*, two species without heteromorphic sex chromosomes, belong to clade C4 [36], a group that also comprises species with highly differentiated sex chromosomes like *C*. *rachovii*, *C*. *vestigipinne*, *C*. *orientale* and *C*. *lanei* ([16], present paper). Considering that several studies had already evidenced the occurrence of an independent origin of sex chromosomes in closely related species and even unusual cases of evolutionary loss of sex chromosomes during the process of species differentiation [37–41], our data reinforces the necessity of a robust phylogeny for the family Crenuchidae as an essential step prior to elaborating a definitive model for the origin of ZZ/ZW sex chromosome system in this group. Moreover, it must be considered that recent studies had already evidenced that *Characidium* is not supported as a monophyletic group [42].

Regardless of the uncertainty about the exact origin of sex chromosomes in *Characidium*, we provided evidence that all sex chromosomes in every analyzed species share amounts of repetitive DNA, since the CgW probe painted at least a small region of all the analyzed Z and W chromosomes, as already described to occur in several other species [10,12–14]. Although these chromosomes present an apparent homologue nature in different species, an extensive interespecific polymorphism related to size, morphology and heterochromatinization degrees can be observed. In addition, our painting results evidenced that some W chromosomes remain highly similar to the CgW probe, while others do not. For example, the W chromosome of *Characidium* sp2 exhibits only a small region of homology with the probe (Fig 4). All these changes reflect the occurrence of several mechanisms that are acting in the differentiation process of these chromosomes, which include chromosomal rearrangements, heterochromatinization, deletion or amplification of segments and differential accumulation of repetitive sequences, as ribosomal genes and microsatellites [9,11,15,17,26].

In general, as demonstrated in other studies, the produced W-specific probes of *C*. *gomesi* hybridize in the centromeric region of the Z chromosome in almost all *Characidium* species [10,12–14]. However, in the present study, the CgW probe hybridized in the subtelomeric regions of the Z chromosome in 2 species: *Characidium* sp2 and *Characidium* sp. aff. *C*. *vidali* (Fig 4). In this sense, although the Z chromosomes are far less susceptible to structural changes than the W chromosomes, some interespecific changes in the Z chromosome in relation to the CgW probe could also be observed in our study, indicating that the Z chromosomes are also subjected to continuous genomic changes during the evolutionary history of *Characidium*. In fact, similar diversification of the Z chromosome among congeneric species was also observed in other fish species [43,44].

Cytological data obtained in the last few years for *Characidium* suggest that the degree of sex chromosomes differentiation in this group would be directly related to the presence or absence of NOR sites in the sex chromosomes. Thus, species with intermediate degrees of differentiation of the ZW chromosomes would present NORs in these chromosomes; while species with highly differentiated sex chromosomes would not [10,14,31]. Remarkably, an exception for this rule is *C*. *vidali*, probably because the NOR translocation is a recent event [15]. In this study, other species might be included in this list, like *C*. *orientale* and *C*. *rachovii*, which present highly differentiated NOR-bearing sex chromosomes and *Characidium* sp2, a species with ZW chromosomes in an intermediate stage of differentiation with 18S rDNA sites only in the W chromosome. Notably, *C*. *orientale* and *C*. *rachovii* presented 5S rDNA sites allocated only in the W chromosome, which could contribute to further decrease the recombination frequency involving Z and W chromosomes and, consequently, favoring the differentiation process of sex chromosomes.

Cytogenetic data obtained in *Characidium* suggest that the isolation of species and populations in headwater components of distinct river basins would be directly related to the elevated rates of chromosome diversification in this genus in both autosomal complement and sex chromosomes [9,10,14,17]. After allopatric differentiation, some species would eventually establish a new contact due to capture events, although they would be already reproductively isolated, as verified to occur with *C*. cf. *zebra* and *C*. *gomesi* from the Upper Paraná River basin, and *C*. *alipioi* and *C*. *lauroi* from the Paraíba do Sul River basin [14].

In the present study, sympatric species were analyzed, including *Characidium* sp. aff. *C*. *vidali* with *C*. *vidali* and *Characidium* sp.5 with *C*. *serrano*, and no interespecific hybrids were identified. Although both sets of species showed the ZZ/ZW sex chromosomes, a low chromosomal differentiation between them was observed, probably reflecting their allopatric diversification before the secondary contact. For example, *C*. *serrano* shows an acrocentric pair while *Characidium* sp.5 does not; and *Characidium* sp. aff. *C*. *vidali* exhibited B chromosomes and *C*. *vidali* did not ([15], present paper). In fact, *C*. *serrano* is a closely related species to the coastal species *C*. *pterostictum*, and its distribution in the Uruguay River basin is related to drainage interchanges that occurred in this area [15,45].

## Supporting Information

S1 Fig karyotypes of *Characidium* species after Conventional Giemsa staining. Bar = 10 μm.
(TIF)

S2 Fig Metaphase chromosome plates after FISH with CgW probe in species without sex chromosome system. Bar = 10 μm.
(TIF)

S1 Table Different *Characidium* species/populations analyzed until now. Highlight the presence/absence of sex chromosome system and the 18S rDNA location.
(DOC)
