# Supplementary figures and images for: Repetitive DNA Sequences and Evolution of ZZ/ZW Sex Chromosomes in Characidium (Teleostei: Characiformes)
Source: PLoS One. 2015 Sep 15;10(9):e0137231. doi: 10.1371/journal.pone.0137231 (PMC4570811; doi:10.1371/journal.pone.0137231)

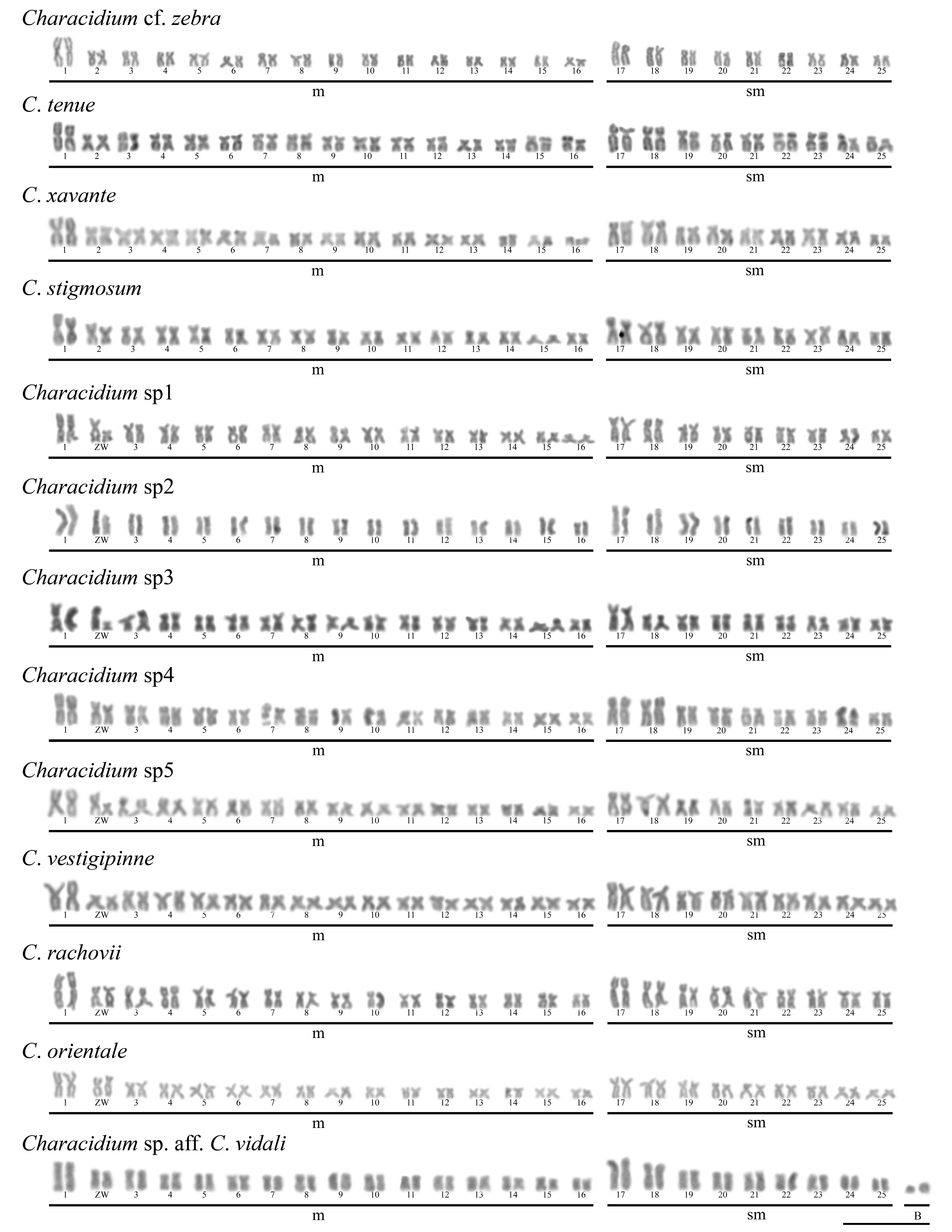

Supplement: S1 Fig — Bar = 10 μm. (TIF) [file pone.0137231.s001.tif]

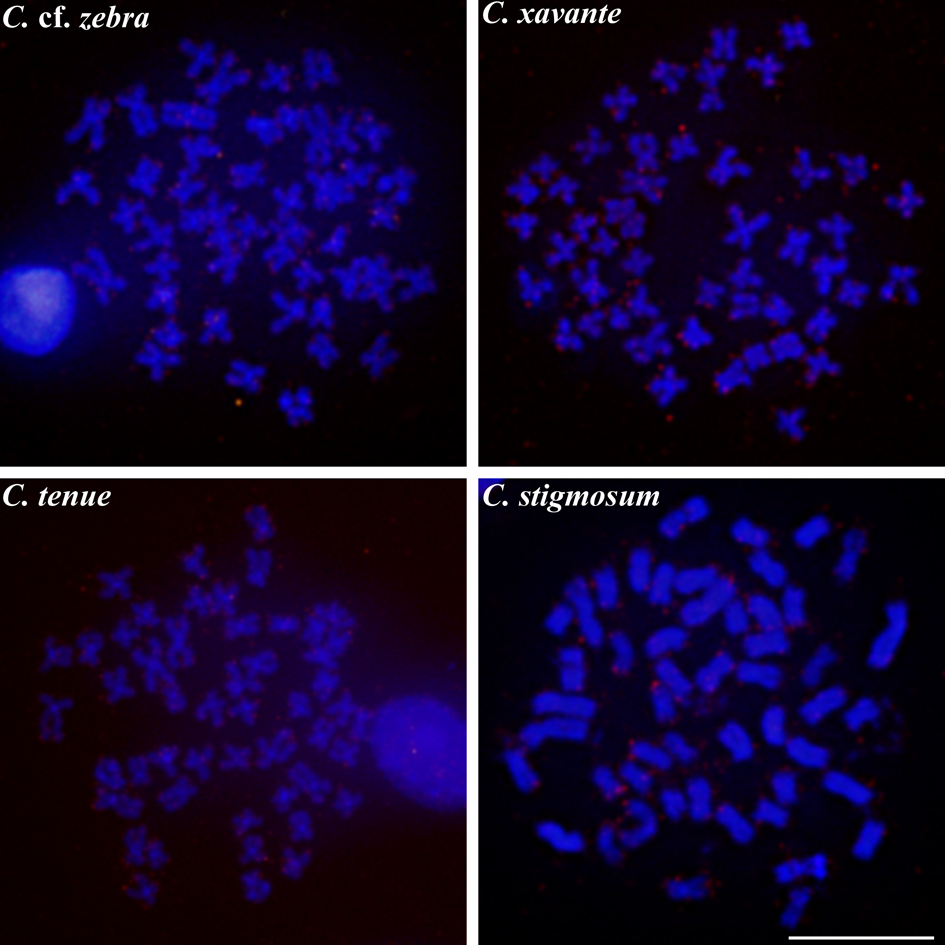

Supplement: S2 Fig — Bar = 10 μm. (TIF) [file pone.0137231.s002.tif]
