# Supplementary material for: Repetitive DNA Sequences and Evolution of ZZ/ZW Sex Chromosomes in Characidium (Teleostei: Characiformes)
Source: PLoS One. 2015 Sep 15;10(9):e0137231. doi: 10.1371/journal.pone.0137231 (PMC4570811; doi:10.1371/journal.pone.0137231)
Supplement: S1 Table — Highlight the presence/absence of sex chromosome system and the 18S rDNA location. (DOC) [file pone.0137231.s003.doc]

**Table S1**- Different *Characidium* species/populations analyzed until now. Highlight the presence/absence of sex chromosome system and the 18S rDNA location.

| **Species** | **Locality** | **Sex Chromosomal ZZ/ZW** | **18SrDNA Present ou**  **Absent in ZZ/ZW** | **Reference** |
| --- | --- | --- | --- | --- |
| *Characidium* cf. *zebra* | Paiol Grande Stream, Sapucaí River basin, São Bento do Sapucaí - SP | Absent | Pair 23 | Centofante et al. 2001 |
| *Characidium* cf. zebra | Machado River, Sapucaí River basin, São João da Mata - MG | Absent | Pair 23 | Silva & Maistro 2006 |
| *Characidium* cf. *zebra* | Pairaitinga River, Tietê River basin, Salesópolis - SP | Absent | Pair 23 | Pansonato-Alves et al. 2010 |
| *Characidium* cf. *zebra* | Pairaitinga River, Tietê River basin, Salesópolis - SP | Absent | Pair 23 | Pansonato-Alves et al. 2010 |
| *Characidium* cf. *zebra* | Alambari Stream, Tietê River basin, Botucatu - SP | Absent | Pair 23 | Pansonato-Alves et al. 2010 |
| *Characidium* cf. *zebra* | Novo River, Pairanapanema River basin, Avaré - SP | Absent | Pair 23 | Pansonato-Alves et al. 2010 |
| *Characidium* cf. *zebra* | Juba River, Paraná River basin, Nova Fernandópolis - MT | Absent | Pair 23 | Pansonato-Alves et al. 2010 |
| *Characidium zebra* | Passa Cinco River, Piracicaba, Ipeúna River basin - SP | Absent | Pair 23 | Machado et al. 2011 |
| *Characidium* cf. *zebra* | Ribeirão Claro Stream, Tietê River basin, Rio Claro - SP | Absent | Not available | Pazian et al. 2013 |
| *Characidium* cf. *lagosantense* | Amendoim Stream, São Francisco River basin, Curvelo-MG | Absent | Not available | Pazian et al. 2013 |
| *Characidium* cf. *fasciatum* | Velhas River, São Franscisco River basin- MG | Present | Not available | Pazian et al. 2013 |
| *Characidium* cf. *zebra* | Duas Antas Stream, Paraguai River basin, Tangará da Serra - MT | Absent | Pair 23 | Present study |
| *Characidium tenue* | Chuí River, Chuí River basin, Chuí - SC | Absent | Pair 23 | Present study |
| *Characidium xavante* | Tributary of Pairanatinga River, Xingu River basin, Pairanatinga - MT | Absent | Pair 23 | Present study |
| *Characidium* *stigmosum* | Tributary of Ave Maria River, Tocantins river River basin, Cavalcante - GO | Absent | Pair 23 | Present study |
| *Characidium* *lauroi* | Tributary of Grande River, Coastal river River basin, Ubatuba - SP | Present | Present | Pansonato-Alves et al. 2010 |
| *Characidium* *lauroi* | Grande Stream, Pairaíba do Sul - SP | Present | Present | Machado et al. 2011 |
| *Characidium* *pterostictum* | Betari River, Betari river River basin, Apiaí - SP | Present | Present | Pansonato-Alves et al. 2010 |
| *Characidium* *pterostictum* | Itapeva Pound, Tramandaí river River basin, Três Forquilhas - RS | Present | Present | Scacchetti et al. 2014 |
| *Characidium* *pterostictum* | Uruguai River Drainage, Cruz Alta - RS | Present | Present | Scacchetti et al. 2014 |
| *Characidium serrano* | Canoinha Stream, Uruguai River basin, Between Pirapó and São Nicolau - RS | Present | Present | Scacchetti et al. 2014 |
| *Characidium* *oiticicai* | Pairaitinga River, Tietê River basin, Salesópolis - SP | Present | Present | Pansonato-Alves et al. 2010 |
| *Characidium oiticicai* | Pairaitinguinha River, Tietê River basin, Salesópolis - SP | Present | Present | Pansonato-Alves et a*l.* 2014 |
| *Characidium lanei* | Barroca River, Atlantico River basin - PR | Present | Present | Noleto et al. 2009 |
| *Characidium lanei* | Cari Stream, Morretes - PR | Present | Present | Pansonato-Alves et al. 2010 |
| *Characidium* sp. | Tributary of Preto River, Itanhaém - SP | Present | Present | Pansonato-Alves et al. 2010 |
| *Characidium* sp. | Formoso River, Pairaná River basin, Chapadão do Céu - GO | Present | Not available | Pazian et al. 2013 |
| *Characidium* sp | Corredeira Stream, Tietê River basin, Poloni - SP | Present | Present | Pucci et al. 2014 |
| *Characidium* *schubarti* | Cinco Réis River, Jaguariaíva River basin, Jaguariaiva-PR | Present | Present | Pansonato-Alves et al. 2010 |
| *Characidium* cf*. gomesi* | Alambari Stream, Tietê River River basin, Botucatu-SP | Present | Present | Pansonato-Alves et al. 2011 |
| *Characidium gomesi* | Alambari Stream, Tietê, Botucatu - SP | Present | Present | Machado et al. 2011 |
| *Characidium* cf*. gomesi* | Cachoeira River, Pairaná River basin, Itumirin - GO | Present | Not available | Pazian et al. 2013 |
| *Characidium* cf*. gomesi* | Novo River, Pairanapanema River basin, Avaré - SP | Present | Pair 18 | Pansonato-Alves et al. 2011 |
| *Characidium gomesi* | Paiol Grande Stream, Sapucaí River basin, São Bento de Sapucaí - SP | Present | Pair 18 | Centofante et al. 2001 |
| *Characidium gomesi* | Machador River, Sapucaí River basin, São João da Mata - MG | Absent | Pair 17 | Silva & Maistro 2006 |
| *Characidium* cf*. gomesi* | Quebra Perna River, Tibagi River basin, Ponta Grossa - PR | Present | 10 Paires marcados | Vicari et al. 2008 |
| *Characidium gomesi* | Minhoca Stream, São Francisco River basin - MG | Present | 18S/terminal/pair 17 | Machado et al. 2011 |
| *Characidium gomesi* | Verde River, Tibagi River basin, Ponta Grossa - PR | Present | Pair 17 e 22 e um dos homólogos do Pair 1 e 20 | Machado et al. 2011 |
| *Characidium* sp. cf*. C. alipioi* | Ribeirão Grande Stream, Ribeirão Grande River basin, Pindamonhangaba-SP | Present | Not available | Centofante et al. 2003 |
| *Characidium heirmostigmata* | Barra Grande River, Ivaí River basin, Prudentópolis - PR | Present | Pair 4 | Pucci et al. 2014 |
| *Characidium timbuiense* | Valsugana Velha Stream, Reis Magos River basin, Santa Teresa - ES | Present | Present | Scacchetti et al. 2014 |
| *Characidium vidali* | Bananeiras Stream, São João River basin, Silva Jardim - RJ | Present | Pair 18 | Scacchetti et al. 2014 |
| *Characidium* sp1 | Russo River, Paraguai River basin, Tangará da Serra-MT | Present | Pair 7 | Present study |
| *Characidium* sp2 | Vermelho River, Paraguai River basin, Tangará da serra - MT | Present | Present no W e um dos homólogos Pair 7. | Present study |
| *Characidium* sp3 | Arinos, Amazon River basin, Nova mutum - MT | Present | Pair 1 | Present study |
| *Characidium* sp4 | Nanay River, Iquitos - Peru | Present | Pair 7 | Present study |
| *Characidium* sp5 | Canoinha Stream, Uruguai River basin - Between Pirapó and São Nicolau - RS | Present | Pair 19 | Present study |
| *Characidium vestigipinne* | Caraguatá River, Uruguai River basin, Coxilha-RS | Present | Present | Present study |
| *Characidium rachovii* | Arroio Cabeças Stream, Uruguai River basin, Rio Grande - RS | Present | Present | Present study |
| *Characidium orientale* | Chasqueiro Stream, Uruguai River basin, Arroio Grande - RS | Present | Present | Present study |
| *Characidium* sp. aff. *C*. *vidali* | Bananeiras Stream, São João River basin, Silva Jardim - RJ | Present | Pair 21 | Present study |
